# Assessing the effective use of mosquito nets in the prevention of malaria in some parts of Mezam division, Northwest Region Cameroon

**DOI:** 10.1186/s12936-016-1419-y

**Published:** 2016-07-26

**Authors:** Ngum Helen Ntonifor, Serophine Veyufambom

**Affiliations:** 1 Department of Biological Sciences, University of Bamenda, North West Region, Bambili, Bamenda, Cameroon; 2 Department of Public Health, Faculty of Health Sciences, Bamenda University of Science and Technology, North West Region, Bambili, Bamenda, Cameroon

**Keywords:** LLINs, Malaria, Prevention, Pregnancy and children 0–5 years, Cameroon

## Abstract

**Background:**

In Cameroon, malaria continues to be endemic and the first major cause of morbidity and mortality among the most vulnerable groups—children under 5 years of age, pregnant women, people living with HIV/AIDS and the poor. The use of long-lasting insecticide-treated bed nets (LLINs) is one of the recommended measures to prevent malaria. The present study was aimed at accessing the acceptability and effective use of LLINs on the prevalence of malaria in PMI Nkwen, Bambui and its environs.

**Methods:**

Hospital-based diagnosis consisted of 476 blood samples that were screened using the rapid diagnostic kits to determine the prevalence of malaria among users and non-users of LLINs. A structured questionnaire was also administered to pregnant women and children less than 5 years of age (476 hospital-based and 350 from the community) which consisted of demographic information, availability, accessibility affordability, acceptability, effective use and problems encountered with the use of LLINs.

**Results:**

Result obtained showed that out of the 476 hospital-based patients, 29 tested positive for malaria giving an overall prevalence of 6.09 %. Equally, results of the questionnaire showed that 743 (89.9 %) of the respondents owned LLINs with up to 649 (87.3 %) having been given to them free-of-charge, and that 578 (77.8 %) were using their LLINs to sleep, even though 18.2 % of the respondents used their LLINs for other purposes, such as fishing, nursing seeds and footfall nets. Malaria was minimal among users of LLINs than non-users and the results were significant at P ≤ 0.05. Also 71.9 % of the respondents said that their nets were in good condition while 52.2 % of them said the major problem with the usage of LLINs was heat and the feeling of suffocation.

**Conclusion:**

These results indicate that LLINs have significantly reduced the prevalence of malaria among the studied population, and so the government should not relent its efforts in the distribution of these nets especially to the vulnerable groups in order eliminate malaria and other mosquito-borne diseases. Utilization of LLINs needs to be encouraged to match ownership, while free distribution of ITNs to vulnerable groups needs to be continuous and consistent.

## Background

Malaria remains the main threat to public health despite decades of control efforts made. It is a devastating disease that threatens productivity and economy of endemic countries [1]. There were over 214 million new cases of malaria worldwide in 2015 and approximately 438,000 malaria deaths [2]. It constitutes over 10 % of Africa’s overall disease burden, accounting for 40 % of public health expenditure, 30–50 % of in-patient hospital admissions and up to 50 % of out-patient visits in endemic areas [3].

Over the last century, efforts have been made to control malaria. Among the new advances in the control of malaria is the use of insecticide-treated nets (ITNs), now mostly long-lasting insecticide-treated nets (LLINs). ITNS are known to kill mosquitoes and have proven repellent properties that reduce the number of mosquitoes that enter the house [3]. They are estimated to be twice as effective as untreated nets [4] and offer greater than 70 % protection compared with no nets [5].

In the past decade, malaria incidence has fallen by at least 50 percent in one-third of the countries where the disease is endemic. These gains have been made through a combination of interventions, including timely diagnosis and treatment using reliable tests and anti-malarial drugs; indoor spraying with safe insecticides; and the use of LLINS to protect people from mosquito bites at night.

Mosquito net ownership is far from universal use despite the aforementioned gains. Ownership rates remain low in many malarious regions or amongst particular groups in malarious regions. Furthermore, mosquito net ownership in itself is not synonymous with utilization. Also ownership is not the only obstacle to achieving the reduction in malaria morbidity and mortality associated with ITN use. Rather, individuals who own (or who have available) mosquito nets must use them in order for the potential health impact to be fully realized [6]. The main strategies for malaria prevention in Cameroon are intermittent preventive treatment (IPT) for pregnant women and vector control through the use of ITNs especially for pregnant women and under-five children [7]. ITNs distributed free-of-charge have been in existence in households in Cameroon since 2003 following the Abuja Declaration in 2000. ITNs are also being distributed in the country free-of-charge to pregnant women during antenatal clinics (ANC) while the rest of the community members obtain their own ITNs from the regional treatment units (RTU) and community treatment units (CTUs) where ITNs are re-impregnated with insecticides after regular intervals of 6 months by community relay agents (CRA), who have been trained to carry out this exercise [2]. While challenges to increasing ITN ownership may diminish as a result of the expansion of large-scale distribution efforts, ITN impact on transmission will be minimized if they are not properly and consistently used, especially among populations vulnerable to increased malaria morbidity and mortality, such as children and pregnant [7].

## Methods

### Study area

The study was carried out in PMI Nkwen, Bamenda (Urban setting) and in Bambui (Rural setting), respectively. Bamenda is located in the North West region of Cameroon. It has a population of high-income earners comprising mostly of civil servants and business men. The population density is high while housing facilities are poor. Bambui is located in the Eastern part of Northwest Region of Cameroon. It is the headquarter of Tubah sub-division, located at 6°3′0″ North, and 10°14′0″ East and about 1350 m above sea level. It has a population of about 50,000 people, with very fertile soils and the majority of its inhabitants are farmers, petty traders and students. These two health areas have common characteristics, such as two seasons (rainy and dry seasons), poor town planning and construction with each room having only one door and one window. Empty plots serve as refuse disposal sites, and defecation places, as most of the bungalows have inadequate or no toilet facilities and no water. In these conditions of very low environmental sanitation and poor personal hygiene, infective diseases abound.

### Sample size and study population

The study population consisted of all patients (pregnant women and children) in PMI Nkwen and Bambui district health centre and respondents in these communities who gave their consent to participate in the study were enrolled. Pregnant women and children included those with or without LLINs.

### Research design

A cross sectional survey was designed to include all children from 0–5 years and pregnant women. The respondents were given questionnaires on their knowledge on the availability, acceptability, effective use of LLINs and the problems faced during the usage.

### Parasitological diagnosis

Thick and thin blood films were prepared, stained with 5 % Giemsa for 30 min and examined under the ×100 (oil immersion) objective of a UNICO^®^ light microscope for the identification of the malaria parasite. Slides were declared negative if no asexual parasites or gametocytes were found after examining 100 high-power fields. For each of the positive slides, parasite density per μl of blood was determined in thick smear on the basis of the number of parasites per 200 leucocytes with reference to participants’ absolute WBC counts [8].

### Statistical analysis

All data collected were entered into SPSS (Statistical Package for the Social Sciences) version 19 (SPSS, Inc, Chicago, IL, USA) for analyses. The frequency of malaria attacks were log transformed before analysis. Associations between the use of LLINs, condition of LLINs, age, how often the nets were washed, education on the use of LLINs, level of education, and malaria prevalence were evaluated using Pearson Chi Square (χ^2^) test. Differences in group means were compared using ANOVA, Student’s *t* test, Mann–Whitney U or Kruskal–Wallis test. Multinomial logistic regression model was used to determine risk factors associated with using LLINs and malaria. Statistical significance was set at P < 0.05.

## Results

### Characteristics of the study population

A total of 826 respondents were recruited for the study out of which 476 (57.62 %) were from the hospital and 350 (42.38 %) were from the community. Out of this lot, 495 (59.9 %) were children aged 0–5 years old while 331 (40.1 %) were pregnant women aged 15–48 years. Respondents that were based in PMI and its environs were 459 (55.57 %) while those that were from Bambui and its environs were 367 (44.43 %). Out of the 476 respondents that were hospital-based, 279 (58.6 %) of children and 146 (30.6 %) of pregnant women had nets while 40 (6.4 %) children and 11 (4.4 %) women did not have.

### Overall prevalence of malaria in the study population

Out of the 476 hospital-based patients, 29 tested positive for malaria giving an overall prevalence of 6.09 %. The prevalence of malaria in children 6.7 % (22/319) was however higher than that of pregnant women 4.46 % (7/157) even though the difference was not statistically significant at P ≤ 0.05. With regards to children, 2.87 % (8/279) who were positive for malaria were using LLINs while 35 % (14/40) were not using LLINs. On the other hand, the seven pregnant women who had malaria were not using LLINs (Table 1).

**Table 1:** Prevalence of malaria in relation to the usage of LLINs amongst children and pregnant women

	Number of patients	Number of patients using LLINs	Number of infected cases		Total	Prevalence of malaria (%)		P value
			Using LLINs	Without LLINs		Using LLINs	Without LLINs	
Children	319	279	8	14	22	2.87	35	0.00
Pregnant women	157	147	0	7	7	0	4.46	0.01
Total	476	426	8	21	29	2.87	39.46	

### Associated demographic data with usage, and like to use LLINs

Respondents were examined in relation to their educational level and the usage of LLINs and from Table 2, 315 (42.3 %) of respondents from Bambui were using LLINs, compared to 430 (57.7 %) from PMI Nkwen. Equally 457 (61.3 %) children aged 0–5 years and 288 (38.7 %) women aged 15–48 years old were using their bed nets. With regards to those who liked using bed nets, 311 (84.7 %) from Bambui liked using LLINs and 443 (96.5 %) from PMI equally liked using them (Table 2).

**Table 2:** Associated demographic information and usage of LLINs

		Usage of LLINs				Like to use LLINs			
		Yes N (%)	No N (%)	Total N (%)	X^2^	Yes N (%)	No N (%)	Total N (%)	X^2^
Village	Bambui	233 (73.9)	82 (26.0)	315 (42.3)	1.6	311 (84.7)	56 (15.3)	367 (44.4)	34.8
	PM I Nkwen	365 (84.9)	65 (15.1)	430 (57.7)	9.9	443 (96.5)	16 (3.4)	459 (55.6)	23.1
	Total	598 (80.3)	147 (19.7)	745 (100)		754 (91.3)	72 (8.7)	826 (100)	
Age	0–5	356 (77.9)	101 (22.1)	457 (61.3)	6.78	478 (96.6)	21 (4.2)	495 (59.9)	46.5
	15–48	242 (84.0)	46 (15.9)	288 (38.7)	14.3	280 (84.6)	51 (15.4)	331 (40.1)	7.02
	Total	598 (80.3)	147 (19.7)	745 (100)		754 (91.3)	72 (8.7)	826 (100)	
Level of education	No schooling	108 (81.8)	24 (18.2)	132 (%)	3.35	127 (88.2)	17 (11.8)	144 (17.4)	0.09
	Primary	144 (62.1)	88 (37.9)	232 (31.1)	0.093	232 (96.6)	8 (3.3)	240 (29.1)	0.41
	Secondary (1st cycle)	102 (97.1)	3 (2.9)	105 (14.1)	6.73	102 (94.4)	6 (5.6)	108 (13.1)	12.7
	Secondary (2nd cycle)	134 (85.9)	22 (14.1)	156 (20.9)	12.39	138 (79.3)	36 (20.7)	174 (21.1)	9.68
	University	110 (91.7)	10 (8.3)	120 (16.1)	5.63	155 (96.9)	5 (3.1)	160 (19.4)	8.60
Total	Total	598 (80.3)	120 (16.1)	745 (100)		754 (91.3)	72 (8.7)	826 (100)	

### Distribution of study population in relation to the frequency of malaria treatment

**Fig. 1:**
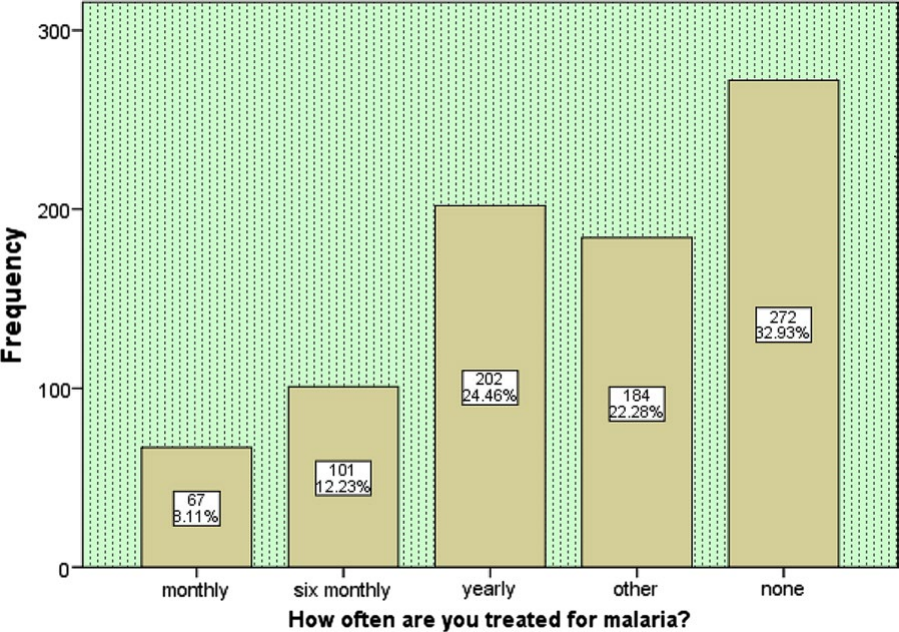
Frequency of malaria treatment among respondents

Out of the 826 respondents, 272 (32.93 %) said they had not been treated for malaria for the past 1 year, 67 (8.11 %) said they were treated monthly while 202 (24.46 %) said they were treated yearly (Fig. 1). According to the respondents using LLINs, 16 (2.34 %) said that they were treated monthly, 43 (7.19 %) almost every 6 months, 133 (22.24 %) yearly, while for those who were not using LLINs, 24 (16.33 %) said they were treated monthly, 41 (27.87 %) six monthly and 43 (29.25 %) yearly. However, 81 (9.8 %) did not respond to the questions (see details on Table 3).

**Table 3:** Frequency of malaria treatment

		Frequency of malaria treatment					Total N (%)	X^2^	P value
		Monthly N (%)	Six monthly N (%)	Yearly N (%)	Other N (%)	None N (%)			
Are using LLINs	Yes	16 (2.3)	43 (7.2)	133 (22.2)	148 (24.8)	258 (43.1)	598 (80.3)	70.3	0.00
	No	24 (16.3)	41 (27.8)	43 (29.3)	28 (18.1)	11 (7.48)	147 (19.7)	36.6	0.00
	Total	40 (5.4)	84 (11.3)	176 (23.6)	176 (23.6)	269 (36.1)	745 (100)		
Condition of LLINs	Very good	0 (%)	15 (3.7)	90 (21.9)	124 (30.2)	181 (44.1)	410 (73.7)	42.1	0.00
	Ok	4 (3.9)	10 (9.7)	18 (17.5)	26 (25.2)	45 (43.6)	103 (18.5)	6.41	0.09
	Poor	12 (27.9)	18 (41.9)	7 (16.3)	4 (9.3)	2 (4.7)	43 (7.7)	2.88	0.41
	Total	16 (2.9)	43 (7.7)	115 (20.7)	152 (27.3)	230 (41.4)	556 (100)		
How often LLINs is used	Everyday	0	15 (3.8)	63 (16.1)	106 (27.1)	207 (52.9)	391 (66.5)	57.9	0.00
	When sick	4 (16)	9 (36)	7 (28)	3 (12)	2 (8)	25 (4.3)	15.9	0.00
	When at home	3 (2.7)	12 (10.7)	47 (41.9)	23 (20.5)	27 (24.1)	112 (19.1)	19.8	0.00
	Not often	10 (16.7)	14 (23.3)	19 (31.7)	9 (15)	8 (13.3)	60 (10.2)	6.50	0.04
	Total	17 (2.9)	50 (8.5)	136 (23.1)	141 (23.9)	244 (41.5)	588 (100)		
Age	0–5	51 (10.3)	58 (11.7)	77 (15.6)	115 (23.2)	194 (39.1)	495 (59.9)	28.9	0.02
	15–48	18 (5.4)	50 (15.1)	123 (37.2)	69 (20.8)	71 (21.5)	331 (40.1)	7.76	0.00
Total		69 (8.4)	108 (13)	200 (24.1)	184 (22.3)	265 (32.1)	826 (100)		

Analysis on how often the respondents were treated for malaria versus the condition of their LLINs was done, with the following results; for those whose net was in good condition, none was treated monthly, 15 (3.5 %) 6 monthly, 90 (21.9 %)yearly, while 181 (44.1 %) said they were not treated at all. For those whose LLINs were in acceptable condition, 4 (3.9 %) were treated monthly, 10 (9.7 %) 6 monthly, and 18 (17.5 %) yearly, while those with LLINs in poor condition 12 (27.9 %) were treated monthly, 18 (41.9 %) 6 monthly, and 7 (16.3 %) yearly (Table 3).

### Accessibility and acceptability of LLINs among respondents

**Fig. 2:**
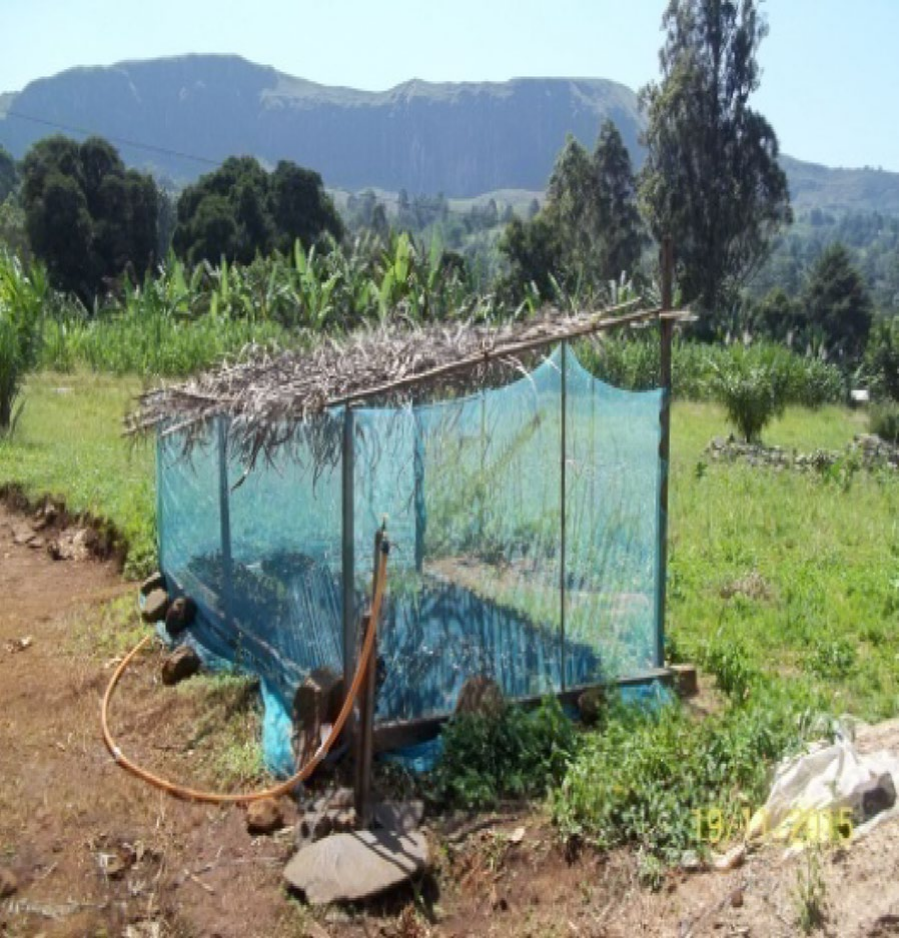
Net used for nursing seeds

The accessibility of LLINs is displayed on Table 4. Out of the 743 respondents that owned mosquito nets, 94 (12.7 %) bought them, while 649 (87.3 %) had been given them free-of-charge by the government. Equally 596 (72.2 %) of the respondents said they could get LLINs from the hospital, while 78 (4.8 %) did not know where to get LLINs from. In their opinion on the cost of LLINs, 226 (27.4 %) said it was cheap, 128 (15.5 %) said it was average, while 21 (2.1 %) said it was expensive and 451 (54.6 %) did not know at all and the results were statistically significant at P ≤ 0.05. With regards to the acceptability of LLINs; 754 (91.3 %) respondents liked using mosquito nets against malaria, while 72 (8.7 %) did not. Out of the 826 respondents, 598 (95.2 %) were using their LLINs and 30 (4.8 %) were not using them, while 198 (24 %) did not answer. Out of the lot that was using LLINs, 578 (96.7 %) were actually using nets to sleep under while 20 (3.4 %) were for other activities, such as football nets, nursing seeds and wall coverings (Fig. 2).

**Table 4:** Accessibility of LLINs among respondents

Source of net	N (%)	Total (%)
Bought	94 (12.7 )	94 (12.7 )
Freely given	649 (87.3)	649 (87.3)
Total	743 (100)	743 (100)
X^2^	14.4	
p value	0.000

### Effective use of LLINs among respondents

The effective usage of LLINs varied among respondents as shown on Table 5. Out of the 826 respondents, 261 (50.2 %) said they use to pull down their LLINs before they got to bed, 158 (30.4 %) fitted them well to the bed before sleeping, 59 (11.4 %) put them permanently over their beds and 42 (8.1 %) of them used nets which were free from holes and could correctly fit the nets over their beds within the right hours. In addition, 374 (71.9 %) of the total population had mosquito nets which were of good conditions, while 306 (37.0 %) did not respond to the question. These results were significant at P ≤ 0.049.

**Table 5:** Effective usage of mosquito nets and the condition of these LLINs

		Condition of LLINs				X^2^	P value
		Very good N (%)	Ok N (%)	Poor N (%)	Total N (%)		
Effective use means	Pulling net down over bed before sleeping	179 (68.6)	52 (19.9)	30 (11.5)	261 (50.2)	6.28	0.39
	Net well fitted on bed in the evening	124 (78.5)	26 (16.5)	8 (5.1)	158 (30.4)	6.06	0.108
	Net putt permanently over the bed	39 (66.1)	15 (25.4)	5 (8.5)	59 (11.4)	14.77	0.022
	Net free from holes and fitted correctly over bed within the right hours	32 (76.2)	10 (23.8)	0 (0%)	42 (8.1)	2.25	0.324
	Total	374 (71.9)	103 (19.8)	43 (8.3)	520 (93.5)		
Age of LLINs (years)	1	240 (58.5)	16 (15.5)	0 (0%)	256 (46.0)	17.1	0.009
	2	130 (31.7)	26 (25.2)	5 (11.6)	161 (28.9)	51.35	0.000
	3	36 (8.1)	35 (33.9)	7 (16.3)	78 (14)	100.5	0.000
	4	4 (0.01)	26 (25.2)	31 (72.1)	61 (10.9)	37.69	0.000
	Total	410 (73.7)	103 (18.5)	43 (7.7)	556 (100)		
How often LLINs is washed	None	318 (91.1)	28 (8.0)	3 (0.85)	349 (62.8)	10.79	0.095
	Once	66 (67.4)	28 (28.5)	4 (4.1)	98 (17.6)	9.97	0.126
	Twice	23 (29.5)	43 (55.1)	10 (12.8)	78 (14)	20.81	0.002
	Often	3 (9.1)	4 (12.1)	26 (78.8)	33 (5.9)	18.07	0.006
Total		410 (73.7)	103 (18.5)	43 (7.7)	556 (100)		

The condition and longevity of LLINs as indicated by the respondents was also analysed. Results showed that 4 (0.09 %) respondents had LLINs that were in good conditions and older than 4 years, 36 (8.8 %) were 3 years old, while 240 (58.5 %) were 1 year old (Table 5). 410 (73.7 %) of the respondents said their nets were in very good condition, while 43 (7.7 %) respondents said that theirs were in poor conditions.

### Problems encountered by respondents using LLINs

Some of the problems surrounding the use of LLINs were analysed. 517 (87.9 %) of the respondents said it was easy putting on LLINs, while 17 (12.1 %) said it was not easy. 307 (52.2 %) respondents said the nets were making them feel hot, while 111 (18.9 %) said they were tired and lazy to put the nets on in the evenings. 91.8 % of the respondents were comfortable with the use of mosquito nets, while only 8.2 % were not.

### Public health awareness on using LLINs in the prevention of malaria

Out of the 826 respondents recruited for the study, only 588 (71.2 %) responded to the public awareness section out of which 527 (89.6 %) said they had been taught on how to use mosquito nets, while for education on the use of mosquito nets in the prevention of malaria, 760 (95.8 %) of the respondents agreed that they had been educated.

## Discussion

The study revealed an overall prevalence of malaria of 6.09 %. These results are in line with those of Sohail et al. [9], who got a prevalence of 5.4 in pregnant women, and to previous reports of 7.9 % by Leke et al. [10], and Mbu et al. [11], who reported 6.6 % of malaria cases among women at routine ANC clinic visits. However, malaria prevalence was less than that reported for out-patient visits by Ndo et al. [12], Egbuche et al. [3], and Anchang-Kimbi et al. [13]. The low prevalence of malaria in this study was probably due to the fact that most of the respondents were pregnant women and children who had bed nets and who were well educated during antenatal visits on the importance of using these nets. Added to this was the fact that the field studies were carried out during the dry season when malaria transmission is at its minimum. *Plasmodium* infections usually increased during the rainy season [14]. The use of LLINs has significantly reduced malaria infection among users as for children. In the present study, it was observed that malaria prevalence in children who were not using LLINs was very high (35 %) compared to those who were using LLINs (2.87 %). The same trend was observed for the pregnant women. This confirms the report of the World Health Organization (WHO) [15], and Ergot et al. [16] on the use of LLINs as a means to reduce the lethal impact of malaria. Malaria occurred at higher prevalence among non-users and this is in line with the works of Ergot et al. [16]. The prevalence of malaria in children was higher than that of pregnant women and this was probably because of the absence of protective immunity in the children and the fact that they could easily roll out of their LLINs at night without their guardians/parents noticing. The respondents whose LLINs were in good conditions were not treated monthly of malaria and 181 (44.1 %) were not treated at all within this period. This shows that the condition of LLINs plays an important role in the prevention of mosquito bites. In addition, respondents who used their LLINs everyday were not treated monthly for malaria and up to 207 (52.9 %) of them were not treated within this period.

The availability of LLINs was very high, most of which had been given free-of-charge by the government. This result is not consistent with the findings of Pulford et al. [6], who explained that ownership rates remained low in many malarious regions or amongst particular groups in malarious regions. Also Kimbi et al. [17] reported that, only 47 % of households interviewed owned at least one mosquito bed net in Buea Health District-Cameroon. LLINs were highly available in these localities because of the effort of the Cameroon government in collaboration with some funding bodies like UN, WHO and UNICEF to achieve the Sixth MDGs by so doing. LLINs are given to all pregnant women during the first antenatal visit to the health centre. In this light one of the major recommendations for malaria control under the Roll Back Malaria initiative in Cameroon since 2013 was free distribution of LLINs to pregnant women and children under 5 years of age [12]. Furthermore, residents of the North West, Centre and East Regions of Cameroon continue to receive free LLINs from other partners, such as PLAN Cameroon (a non-governmental organization).

LLINs were equally accessible to the people as 90.6 % of the respondents knew where to get the nets from. In addition to this, most of the respondents did not know how much LLINs could cost probably because they were used to free donations. This confirms the WHO measures [18] to reduce malaria morbidity and mortality through large-scale programmes of distribution of free or highly subsidized LLINs, which lastly took place in October 2015 in the North West and South West Regions of Cameroon. Despite the fact that many did not know the cost of LLINs, the few that knew were of the opinion that the nets were cheap making them affordable to those who missed the chance to receive them.

The majority (79.3 %) of the respondents were of the opinion that LLINs can help to prevent malaria and with regards to the acceptability of LLINs; 91.3 % of the respondents liked using mosquito nets against malaria. This is in line with the WHO [18] and Lengeler [1], who stated that on control measures to reduce malaria, the most promising measure is the use of ITNs and curtains. These two factors actually contributed to the high and appropriate use of LLINs among owners as 93.0 % of respondents were using their bed nets to sleep under. These findings are not in conformity with those of Pulford et al. [6] who observed that between 15–50 % of available nets were unused in Nigeria. Also the level of education of the respondents played a significant role in the usage of LLINs because most of the respondents were actually adults with at least a secondary school level of education.

Some studies hold that, mosquito net ownership in itself is not synonymous with utilization [6, 19]. This is not the case with the findings in this study as most of the respondents that had nets were using them. However, out of those that were using LLINs, 96.7 % were actually using them to sleep under while 3.4 % of the respondents were using them as football nets, nursing seeds, and wall cover, harvest bean or to catch fish. This high usage of LLINs among respondents in this locality may be due to public health awareness of the dangers of malaria attack in the studied population and the emphasis on the importance of LLINs to reduce the prevalence of malaria during antenatal and postnatal clinics.

The meaning of effective use varied between respondents. However, whatever the meaning, the important thing is that the LLINs were being used and this offers a degree of protection against the female Anopheles mosquito biting because they bite mostly in the night.

The age of LLINs, how often it has been washed and its general condition is one of the main cardinal factors in the effectiveness of LLINs. This is because even if the LLINs are fitted correctly over the bed within the right hour, old ones with many holes greater than 10 cm and in which the deltamethrin or permethrin chemical has been washed out will not offer the protection that it is supposed to do since mosquitoes can land on it and bite through the fine holes or go directly into the net. Bachou et al. [5] estimated that LLINs offer greater than 70 % protection compared with no nets. The findings of this study showed that most of the respondents had nets that were in good conditions, and also most of them had not washed their LLINs. All these factors contributed to the effectiveness of their LLINs in the prevention of mosquito bites and the low prevalence of malaria. This roughly supports the report of Yakob and Guiyun [20], which stated that mathematical modeling, has suggested that disease transmission may be exacerbated after bed nets have lost their insecticidal properties under certain circumstances. In this case, since the protection of the LLINs was high, the prevalence of malaria was low.

Some of the respondents in the study said that using LLINs daily was boring and most of the time they were tired and lazy to use them. Others claimed that they did not like using nets because of the bad smell, boring routine exercise of putting them up and down, and the feeling of suffocation. Heat was one of the main problems respondents encountered that could make them not to use LLINs at times, as up to 52.2 % of the respondents said the nets usually gave them heat. This is in line with the reports of Pulford et al. [6].

Education of individuals as well as communities plays a fundamental role in the control/eradication of malaria. From the present study, a strong factor that has reduced malaria cases among the children and pregnant women is education on how to prevent malaria at the primary health care level. This has widened the scope of activities of respondents on keeping the environment clean, clearing bushes around, avoiding standing water in containers, wearing protective clothing and taking anti-malarial drugs.

## Conclusions

Pregnant women and guardians need to be sensitized to purchase ordinary bed nets and take them for impregnation at the CTUs if they do not have any. CRAs activities also need to be updated to involve hanging of nets in homes and helping household members to overcome social and personal barriers in the use of bed nets.

## Abbreviations

ANC: antenatal care clinic
LLINs: long-lasting insecticide-treated bed nets
ITNs: insecticide-treated nets
